# Efficiency of Bacteriophage-Based Detection Methods for Non-Typhoidal *Salmonella* in Foods: A Systematic Review

**DOI:** 10.3390/v16121840

**Published:** 2024-11-27

**Authors:** Preeda Phothaworn, Chatruthai Meethai, Wanchat Sirisarn, Janet Yakubu Nale

**Affiliations:** 1 Center of Excellence Research for Melioidosis and Microorganisms (CERMM), Walailak University, Nakhon Si Thammarat 80160, Thailand; preeda.ph@wu.ac.th; 2 Department of Medical Technology, School of Allied Health Sciences, Walailak University, Nakhon Si Thammarat 80160, Thailand; chatruthai.me@wu.ac.th; 3 Department of Microbiology, Faculty of Medicine, Kasetsart University, Bangkok 10900, Thailand; wanchat.s@ku.th; 4 Centre for Epidemiology and Planetary Health, School of Veterinary Medicine, Scotland’s Rural College, Inverness IV2 5NA, UK

**Keywords:** bacteriophages, bacterial detection, food pathogens, *Salmonella enterica*, *Salmonella* diagnostics, gastroenteritis, food safety

## Abstract

Food contamination with non-typhoidal *Salmonella* (NTS) presents a significant public health risk, underscoring the critical need for rigorous food safety measures throughout the production, distribution, preparation, and consumption stages. Conventional diagnostic strategies are time-consuming and labor-intensive and are thus sub-optimal for throughput NTS detection. Bacteriophages (phages) are highly specialized bacterial viruses and exhibit extreme specificity for their hosts. This organic phage/bacterial interaction provides an invaluable tool that can potentially replace or complement existing *S. enterica* detection methods. Here, we explored work in this area and reviewed data from PubMed/MEDLINE, Embase, and ScienceDirect up to 4 November 2024. Thirty-five studies were selected from 607 retrieved articles using the JBI Critical Appraisal Checklist to ensure quality. *Salmonella* enrichment, rapid detection, and effective recovery in diverse food sources for various NTS serovars were targeted. Utilizing phages as bio-probes alongside lateral flow immunoassays, surface-enhanced Raman spectroscopy, fluorescence, and electrochemistry assays enabled rapid and highly sensitive detection of NTS, achieving limits as low as 7 to 8 CFU/mL within 30 min. Balancing detection sensitivity with rapid analysis time is essential. Further research and development will be pivotal to overcoming challenges and maximizing the efficiency of NTS phage-based detection to ensure optimal food safety.

## 1. Introduction

Non-typhoidal *Salmonella* (NTS) is among the most prevalent and prioritized bacterial pathogens and causes approximately 150 million gastroenteritis and 60,000 deaths in humans annually from food-borne, water-borne, and zoonotic infections worldwide [1,2,3]. Approximately 2500 serovars of *S. enterica* have been identified to cause the infection in various vertebrates, but only ~100 of these serovars are linked to outbreaks in humans [4]. The approximately 100 NTS serovars implicated in human infections are derived from the combinations of 46 O-antigens and 114 H-antigens. Among these, the serovars O:4, H:i:1,2 (*S*. Typhimurium) and O:9, H:g,m:1,7 (*S*. Enteritidis) are particularly significant due to their high prevalence and clinical importance [5]. These antigens play a dual role, functioning as markers for serovar classification as well as key factors in interactions with external agents, including antibodies and bacteriophages (phages, viruses that specifically target and infect bacteria) [5].

The diversity of NTS serovars and their niche dynamics highlight their public health importance, requiring strain-specific surveillance strategies and control measures. For this reason, highly sensitive and effective detection mechanisms of the pathogen in various sample sources is critical.

Foods such as pork and poultry products remain the highest-risk sources of NTS infection [6]. However, occasionally, *Salmonella* infection is also found to be associated with rarely reported food sources such as chocolate, which was linked to the largest recent outbreak in the EU/EEA, with 15.5 cases detected per 100,000 population in 2022 [7]. Furthermore, *Salmonella* is recognized as the most commonly detected foodborne pathogen in imported foods in the EU, with the majority (64%) of the notifications being linked to sesame seeds outsourced from Africa [8]. NTS is also a major cause of foodborne illnesses in Southeast Asian countries like Thailand, Vietnam, and Malaysia, with outbreaks frequently linked to undercooked poultry, eggs, seafood, and street food [9]. These reports clearly indicate the diverse and widely variable sources of the infection, requiring effective detection strategies in all food processing systems and sources.

Although NTS infections in healthy individuals often result in mild and self-limiting symptoms, they can lead to more complex and severe manifestations in vulnerable populations, such as infants, the elderly, and other immunocompromised individuals, than in healthy groups [9,10,11]. The detection of NTS contamination in food supply chains is therefore extremely crucial for overall public health safety and to prevent disease outbreaks.

Various methods for *Salmonella* detection exist, including immunological- and molecular-based techniques, mass spectrometry, spectroscopy, optical phenotyping, and biosensor methods [12]. The electrochemical biosensor is particularly gaining more traction for *Salmonella* detection than the aforementioned methods due to its high sensitivity, speed, and portability. However, despite this advancement, specific bioreceptors like aptamers, antibodies, and phages, along with nanomaterial applications, are still required to enhance the effectiveness of these strategies [12,13,14]. While phage-based detection systems may employ signaling mechanisms similar to those used in antibody- or aptamer-based methods, they exhibit the ability to distinguish between variable bacterial strains, offering greater specificity and versatility in detecting a broad range of bacterial pathogens [15]. The extensive serovar diversity within *Salmonella* presents considerable challenges for antibody- or aptamer-based detection methods. These approaches are typically limited to recognizing only specific subsets of *Salmonella* serovars, which reduces the effectiveness of separation techniques in detecting the full genus. Also, these conventional methods for detecting *Salmonella*, as well as culture-based techniques, whilst reliable, are often time-consuming, labor-intensive, and unsuitable for throughput analyses [16]. Consequently, there is a growing demand for more rapid, sensitive, and specific strategies for optimum *Salmonella* detection in all food sources and safety management systems to replace or supplement existing methods.

Phages have emerged as a promising tool in the detection and control of bacterial pathogens, including *Salmonella* [17,18]. *Salmonella* detection using innovative phage technology leverages the natural specificity and binding affinity of phages to their bacterial hosts, thus enabling the development of highly selective and sensitive detection assays. This method potentially offers several advantages over conventional *Salmonella* detection techniques, including shorter detection times, reduced need for sample preparation, and the capability for on-site testing [19,20]. Crucially, phage-based methods precisely target live bacteria, effectively distinguishing viable cells from non-viable ones to ensure the detection of active infection. In contrast, molecular techniques (for example PCR) can detect very low levels of genetic material but usually necessitate additional steps to differentiate between live and dead cells. The use of phages for detecting *Salmonella* in food has been explored in various studies, employing different phage-based approaches such as targeting whole phage amplification [21], expression of phage-encoded reporter genes, and detection of phage-mediated bioluminescence [22,23,24]. However, the efficiency and reliability of these methods can vary significantly depending on the type of food matrix, the specificity of phages used (whether narrow or broad host range phages), and the detection protocols applied [25].

A recent review in this field focused on a limited selection of studies, narrowing down to just eleven studies that explored the use of phage-based sensors for detecting *Salmonella* spp. and their application in five different food samples [26]. To further knowledge in the area and expand beyond the limited studies above, here, this systematic review was carefully designed to comprehensively evaluate the current state of phage-based detection methods for NTS serovars in various food sample types. To achieve this, we conducted three searches in PubMed/MEDLINE, Embase, and ScienceDirect of research studies in the area up until 4 November 2024 and screened the output against a list of inclusion and exclusion criteria. The resulting data was synthesized to present the overall efficiency, sensitivity, and specificity of the phage-based detection methods identified for each serovar and food type. Furthermore, factors which play key roles in the overall performance of the methods were also established, and mitigation steps to improve effectiveness were highlighted and discussed. Our work provides valuable insights into the potential of phage-based detection as a viable alternative to conventional methods in food safety diagnostics and management, using NTS as a model.

## 2. Materials and Methods

This systematic review was conducted to evaluate the effectiveness of phage-based detection methods for NTS serovars in food samples. The study followed the guidelines outlined in the Preferred Reporting Items for Systematic Reviews and Meta-analyses (PRISMA) 2020 statement [27]. The review was not registered due to time constraints and the extensive number of studies being conducted in this area.

### 2.1. Data Search Strategy and Extraction

A comprehensive search was carried out in three databases, namely PubMed/MEDLINE, Embase, and ScienceDirect, to identify relevant literature from their inception dates up to 4 November 2024. The search strategy for each database and the number of studies searched were based on the terms provided in Supplementary Tables S1–S3. Duplicate records were removed using both EndNote 21 tool [28] and manual screenings. Only studies written in English were considered for inclusion. Three reviewers independently screened the titles and abstracts of all retrieved records from the database search to determine eligible studies using Rayyan review manager [29]. The reviewers retrieved and screened the full-text articles according to the pre-specified inclusion and exclusion criteria as highlighted below. All studies chosen to be excluded were discussed, and the reasons for the exclusion were recorded. All retrieval processes were performed manually and independently by three researchers. Any disagreement during the selection process was resolved by consulting a fourth reviewer.

### 2.2. Selection Criteria of Studies to Be Included in This Systematic Review

Eligibility criteria were designed based on the PICO acronym [30] for the research question: Population: foods contaminated with NTS; Intervention: phage-based detection methods; Comparison: among all phage-based detection methods we identified; and Outcome: efficiency of detecting NTS in food samples. To ensure adherence to our research objectives, we implemented specific eligibility criteria for study inclusion. Only research articles published in English were considered for analysis. We gave precedence to studies investigating the detection of non-typhoidal *S. enterica* in food samples, encompassing research on both whole phage particles and phage component-based methods. Prioritizing study on NTS detection is critical for protecting public health. This is to ensure food safety and advance scientific expertise and technology in foodborne pathogen detection and management. Studies falling into categories such as reviews, mini-reviews, comments, or editorials were excluded. Additionally, studies involving environmental and clinical samples, pure non-typhoidal *S. enterica* cultures, typhoidal *S. enterica*, or strictly other food pathogens were excluded from our analysis.

### 2.3. Quality Assessment and Methodological Evaluation

Following rigorous scientific protocols, the methodological quality of the included studies was assessed using the JBI Critical Appraisal Checklist for Systematic Reviews [31]. Two independent reviewers (P.P. and C.M.) carefully evaluated each study based on four adopted critical questions. These items included: “Is the review question clearly and explicitly stated?”; “Were the inclusion criteria appropriate for addressing the review question?”; “Did the study provide fully available details of data?”; and “Was the measurement of the intervention conducted with validity and reliability?” Criteria indicating low methodological quality included one or more “no” answers or “unclear” responses to critical questions or three or more “no” answers to non-critical items. High methodological quality was determined when a study had a maximum of two “no” responses and one “unclear” response in non-critical items. Studies not meeting these criteria were considered to have moderate methodological quality (Supplementary Table S4).

### 2.4. Analysis of Data

The systematic review was conducted using a pipeline and manual screening, following the PRISMA method. However, due to significant heterogeneity among the included studies, conducting a meta-analysis was deemed unfeasible. The studies and their results were grouped and analyzed based on the stage of phage implementation for NTS detection, either during bacterial enrichment or the final stage of bacterial detection, the efficiency of the detection methods based on the rapid phage detection platforms used, types of food samples, limits of bacterial load, and specificity and sensitivity of the assay.

## 3. Results

**Figure 1:**
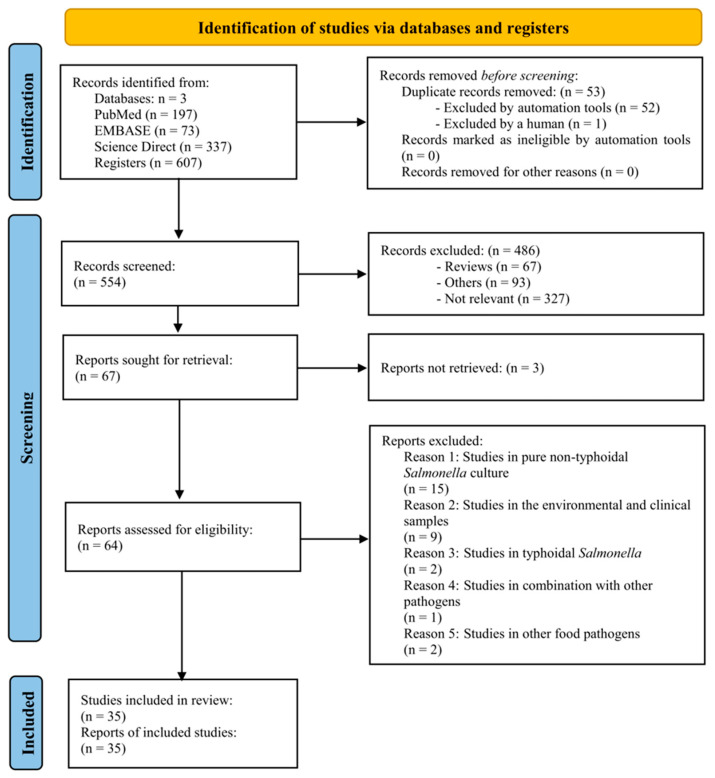
PRISMA flow diagram to visually represent the process of screening and selecting articles used in this systematic review. Comprehensive database searches yielded a total of 607 records, sourced from PubMed/MEDLINE (197 records), Embase (73 records), and ScienceDirect (337 records). After applying the inclusion and exclusion criteria, 35 English-language documents were selected for systematic review analysis.

In this systematic review, we identified the pertinent literature on the application of phages to detect NTS in all food sample types. To accomplish this, we commenced with keyword searches across scientific research records and databases, as shown in Supplementary Tables S1–S3. These searches yielded a comprehensive pool of 607 records sourced from the prominent platforms PubMed/MEDLINE, Embase, and ScienceDirect, with 197, 73, and 337 records, respectively. Subsequently, 35 English-language documents were meticulously handpicked for in-depth scrutiny by a panel of three independent reviewers based on our inclusion and exclusion criteria as stated above (Figure 1). To ensure rigor and consensus in the selection process, a fourth reviewer was engaged as an arbiter in cases of discordance.

### 3.1. Distribution of the Selected Studies

**Figure 2:**
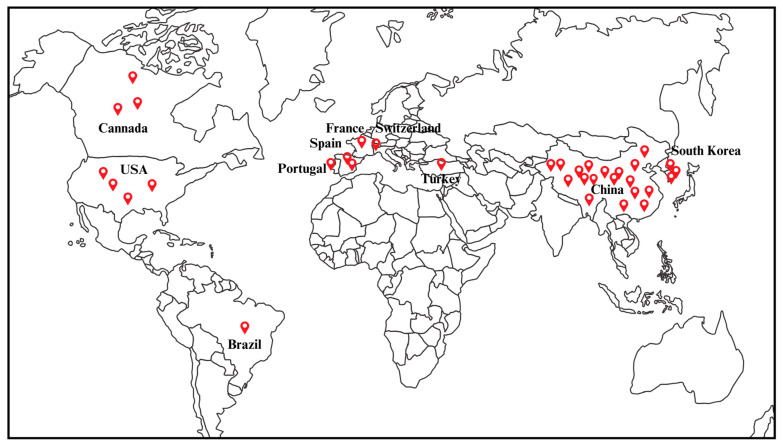
Visualization of the distribution of studies on phage-based detection of non-typhoidal *Salmonella* in food samples based on the country of publication as identified in this systematic review.

The geographic distribution of studies on phage-based detection for NTS in foods is illustrated in Figure 2. Approximately, half of the selected publications, approximately 51.43% (18 out of 35), originated from China. This is followed by 11.43% (four out of thirty-five) from the USA, 8.57% (three out of thirty-five) each from Canada and South Korea, 5.71% (two out of thirty-five) from Spain, and 2.86% (one out of thirty-five) each from Brazil, France, Portugal, Switzerland, and Turkey. This systematic review reveals that phage-based detection methods for NTS in foods have been in development since 1983, continuing to the present day. Notably, the highest number of research articles in a single year was published in 2023, accounting for eight out of the thirty-five articles. There has been a notable increase in research activity per year after 2020.

### 3.2. Concepts of Phage-Based NTS Detection in Food Safety Testing

Phage-based bacterial detection methods, particularly when combined with enrichment steps, offer a promising and rapid alternative to traditional bacterial detection methods in food safety testing. Their high specificity and ability to rapidly detect live bacterial cells by infecting and replicating within their hosts make phage-based assays an attractive option for detecting active infection, ensuring food safety and preventing foodborne illnesses [32]. However, whole phage particles have limitations as biorecognition elements. Their lytic activity can disrupt signal stability, and random immobilization often reduces sensitivity and bacterial capture efficiency [33,34]. Additionally, their large size can impair signal sensitivity in distance-dependent systems [35]. Phages recognize bacterial hosts through receptor-binding proteins (RBPs) such as tail fiber proteins (TFPs) and tail spike proteins (TSPs), which mediate adsorption and injection. RBPs are advantageous for their high sensitivity and specificity, stability without inducing bacterial lysis, compact structure, and ease of production via recombinant methods [36]. Consequently, RBPs exhibit ideal molecular recognition properties, combining high specificity with strong resistance to interference from non-target bacteria.

**Figure 3:**
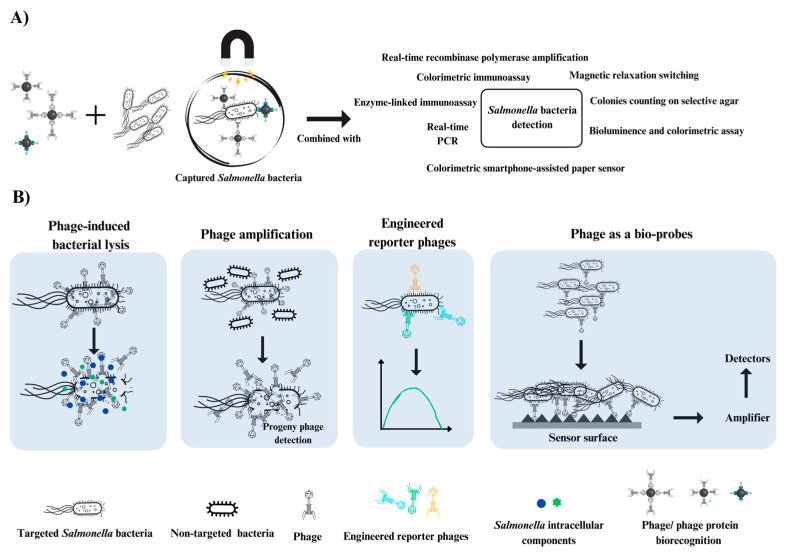
Schematic representation of the combination of different methods for capturing and detecting *Salmonella* bacteria. The figure shows the binding of targeted *Salmonella* bacteria (depicted as a rod-shaped structure) by specific agents, followed by capture using magnetic beads. The detection techniques include colorimetric immunoassays, real-time PCR, enzyme-linked immunoassays, magnetic relaxation switching, colorimetric smartphone-assisted paper sensors, and colony counting on selective agar (**A**). Detailed mechanisms of phage-based detection: phage-induced bacterial lysis, phage amplification, engineered reporter phages, and phages as bio-probes (**B**).

Generally, applications of phages as tools for bacterial detection in food can be categorized into two major purposes: enriching or capturing pathogens from food matrices and utilizing their specificity to detect pathogens (Table 1). Based on these reasons, the selected studies here were categorized into two subgroups: *Salmonella* enrichment and *Salmonella* detection, as discussed below (Figure 3). The quality assessment of each study is summarized in Table 2.

#### 3.2.1. *Salmonella* Enrichment

Enrichment is a necessary step in the detection of *Salmonella* in food samples due to the low numbers of bacteria typically present, the potential for bacterial injury, competition from other microorganisms, and regulatory requirements [68,69,70]. Scientific studies and regulatory standards support the use of enrichment to improve the sensitivity and reliability of *Salmonella* detection methods. This challenge can be addressed using high-affinity biorecognition reagents that bind to target pathogens [71]. The combination of magnetic microbeads (MBs) and recognition elements, such as specific phages and aptamers, was used to recognize and separate pathogens [72].

Here, eight out of the thirty-five included studies reported the efficiency of *Salmonella* captured from 12 types of food samples: pork, chicken meat, lettuce, skim milk, orange juice, eggs, milk, fresh milk, chocolate milk, infant formula, celery, and alfalfa sprouts. The recovery efficiency ranged from 65% to 102%, with an initial artificially inoculated bacterial concentration of approximately 10^5^ CFU/mL (Table 2), and each method did not discriminate between solid and liquid food samples.

Magnetic microbeads (MBs-COOH) or magnetic nanoparticles (MNPs-COOH) were commonly used to separate the bacteria from the food matrix. The principle of separation entails immobilizing magnetic beads (MBs) with biorecognition molecules like antibodies, and phage proteins, which possess high selectivity for target analytes. Both intact phage particles and phage-derived bacterial binding proteins (PBPs) serve as effective biorecognition elements for the capture of bacterial pathogens. The remarkable binding specificity of phages and PBPs offers significant potential for applications in bacterial diagnostics and monitoring, spanning fields such as food safety, environmental surveillance, and healthcare [73]. The phage-derived tail spike protein (TSP) from phage P22 [42] demonstrated superior capture efficiency for *S*. Typhimurium, achieving rates exceeding 90% and outperforming antibodies, which achieved 80% capture efficiency [74]. However, the mechanism of how the phage interacts with *Salmonella* binding protein is unclear. In addition, the streptavidin-coated MBs conjugated with the specific long tail fiber (LTF) protein of phage S16 can increase the capture efficiency to 98.00% ± 1.00, with an initial bacterial concentration range of 10^1–5^ CFU/mL [22].

To optimize the capturing efficiency of NTS, the immobilization efficacy of phages or their proteins on magnetic beads was tested. Phage P22, a member of the *Podoviridae* family, achieved a higher coupling efficiency with magnetic microparticles (92.40%) than with nanoparticles (83.80%) [61]. When comparing the coupling efficiency of different magnetic nanoparticles of varying diameters (1000 nm, 250 nm, 180 nm, and 150 nm), the results indicated that larger magnetic nanoparticles allowed more phages such as LPST10, belonging to the *Siphoviridae* family, to immobilize. However, there was no significant difference in the capturing efficiency of the phage-magnetic conjugates for *Salmonella* [50].

Additionally, various concentrations of phages or phage components were tested. In the P22-MBs covalent immobilization process, the number of phages per MBs increased significantly, plateauing at a phage/MB ratio of approximately 1650, with up to 2.0 × 10^11^ phages immobilized on 7.0 × 10^7^ MBs. To assess the impact of varying phage receptor- binding protein (RBP) 41 concentrations on the capture efficiency of the probe, different amounts of RBP 41 were coupled with MBs. Like the immobilization of whole phage particles with MBs, the capture efficiency of the RBP 41 for *Salmonella* increased with higher concentrations of the protein. A concentration of 115 µg/mL RBP 41 was found to be optimal, achieving a capture efficiency of approximately 83.43%. There was no significant difference in capture efficiency between 115 µg/mL and 1150 µg/mL of RBP 41 coupled MBs [47]. The bacterial capture efficiency was influenced not only by the size of the MBs and the concentration of phages or phage proteins but also by several key parameters optimized to maximize the binding efficiency of *Salmonella* cells. These parameters included the single-reaction volume, the incubation time with *Salmonella* culture, the pH value of the reaction solution, and the NaCl concentration in the reaction solution.

Further studies ascertained the optimal conditions for coupling phage proteins such as the long tail fiber proteins of STP4-a (LTF4-a) with MNPs to be 0.1 mg/mL protein solution per reaction, in a neutral to weakly alkaline environment (pH 7.5), with an incubation time of 30 min at room temperature. The T4-like phage, STP4-a, utilizes lipopolysaccharide (LPS) and outer membrane proteins as primary receptors, enabling high infectivity and a broad host range, successfully targeting 88 out of 91 *Salmonella* strains across 24 serovars [75]. These conditions achieved optimal recovery efficiency, supporting rapid and effective *Salmonella* detection [51].

Instead of solely relying on phages or phage components for capturing and enriching targeted NTS in food samples, they could also be employed as dual probes. To achieve this, composite probes were developed that consisted of deactivated phage-modified magnetic beads (MB-P) and PtNP-antimicrobial peptide (Pt-AMP) nanozyme probes. These probes form sandwich complexes with *S*. Typhimurium, emitting both bioluminescent and colorimetric signals for live and total *Salmonella* cells, respectively, and these can be simultaneously measured on a single microfluidic platform [40].

The validated concepts for using specific phages or phage proteins as bio-probes to capture *Salmonella* from food matrices are summarized in Figure 3A. Bacterial recovery or enrichment is the initial step in bacterial detection and must be combined with other methods to identify bacterial cells in samples. These methods include colony counting on selective agar, enzyme-linked immunoassay, and real-time PCR, as discussed below.

#### 3.2.2. *Salmonella* Detection

Phage-based methods for food bacterial detection utilize phage specificity and rapid replication, providing evidenced advantages. As indicated above, this strategy ensures accurate detection in food samples by targeting only viable bacteria. Virulent phages complete their infection cycle within approximately 1–2 h, and this speeds detection time compared to traditional methods [76]. Moreover, phages are stable in a wide range of environmental conditions (temperature, pH, organic solvents), ensuring reliable detection across diverse food matrices and processing conditions [26]. Based on these advantages, phages have shown great potential for *Salmonella* detection in food, through targeted phage-induced bacterial lysis, phage amplification, genetically modified reporter phages, and phage biosensors, as shown in Figure 3B and discussed below.

1. *Phage-induced bacterial lysis*

The final step in the life cycle of lytic phages is the lysis from within of the host cell, leading to bacterial cell destruction. This process releases phage progeny and intracellular components, such as adenosine triphosphate (ATP), intracellular enzymes like adenylate kinase (AK) and caprylate esterase, and cytoplasmic components like β-D-galactosidase, which can serve as markers for detecting the lysis event [76,77]. Caprylate esterase is an enzyme that catalyzes the hydrolysis of caprylate esters, resulting in products that can be easily detected through various assay techniques.

In the context of phage-based detection, a recent study by Zhang et al. (2023) demonstrated the use of caprylate esterase as a detection marker for *S*. Enteritidis in milk. In this study, *Salmonella* cells lysed by phage LPSE1 released caprylate esterase, and the product of this reaction, salicylaldazine, was detected using a fluorescence assay [44]. When combined with pre-enrichment of *Salmonella* cells using ampicillin-conjugated magnetic beads, the developed method offers high accuracy, with a broad detection range (10–10^6^ CFU/mL), a low detection limit (6 CFU/mL), and a short detection time (within 2 h) for rapid *S*. Enteritidis detection in milk.

Continued research and development in the area of utilizing caprylate esterase as a marker for bacterial pathogens is beneficial in four aspects: (1) Specificity: phages and bacterial cells are highly specific in their binding interactions. Phages typically recognize receptors on the bacterial cell surface with high precision, which is advantageous for targeting specific *Salmonella* serovars. The specificity of binding largely depends on the structural compatibility between the phage binding proteins and the target bacterial surface molecules. The application of phages to recognize caprylate esterase released from specific targeted bacterial cells can minimize false positives. (2) Sensitivity: phages generally provide a broader and more adaptable binding range, which enhances sensitivity especially in complex or challenging environments. Fluorescence assays can detect even small amounts of the reaction product, allowing for the identification of low levels of bacterial contamination. (3) Speed: due to their natural evolutionary design, phages bind quickly to their specific bacterial hosts. This rapid recognition often allows for a faster initial attachment to the target bacteria in a sample, particularly when high-affinity wild-type phages are used. (4) Simplicity: compared to culturing *Salmonella*, using phages is a lot easier, and the assay does not require sophisticated equipment, making it accessible for various laboratory settings and needs. Despite its advantages, the use of caprylate esterase as a marker also faces several challenges. These challenges include ensuring enzyme stability under various environmental conditions, maintaining phage specificity to minimize cross-reactivity with non-target bacteria, and optimizing the assay to ensure consistent performance [78].

In addition to caprylate esterase, adenosine triphosphate (ATP) is also used as a marker for detecting NTS. Wang et al. (2024) developed a platinum nanoparticle- decorated phage 2-nanozyme (P2@PtNPs) for sensitive, on-site detection of live and dead *S.* Typhimurium. The detection limits for this are 30 CFU/mL for total bacteria and 40 CFU/mL for live bacteria. Recovery rates for spiked pork, fish, and milk ranged from 91% to 108% [41]. To achieve a lower limit of detection of 20 CFU/mL, a phage-assisted DNA and ATP synergistically triggered Argonaute-mediated fluorescent (PDAAF) biosensor for DNA extraction- and amplification-free detection of viable *S*. Typhimurium with the phage LPST10. Spiked recoveries in chicken meat samples ranged from 87.72% to 113.97% [42]. This rapid and versatile assay offers a reliable, cost-effective tool for assessing bacterial vitality and enhancing food safety diagnostics.

2. *Phage amplification*

**Figure 4:**
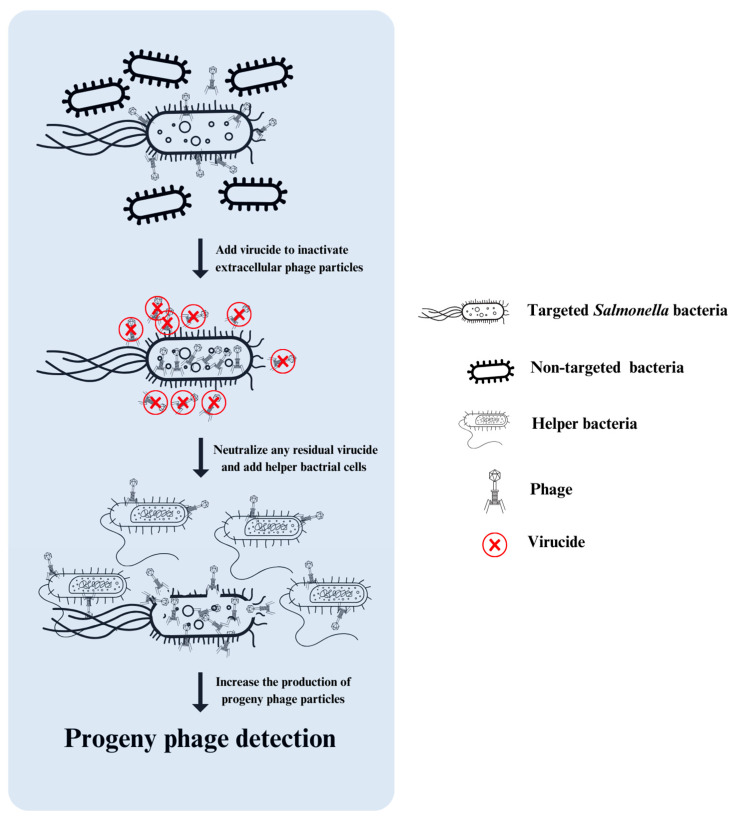
The principle of phage amplification assay (PAA) for bacterial detection. The process starts with (1) inoculation: mix the sample with specific phage and incubate under suitable conditions to allow phages time to infect any target bacteria present; (2) virucide treatment: add a virucide to the mixture to inactivate extracellular (unbound) phages; (3) helper cell addition: introduce helper cells of a bacterial strain that supports phage replication to the sample as a propagation strain; and (4) progeny phage detection: measure the presence of progeny phages through plaque formation on agar or by identifying phage nucleic acids.

Phage amplification is the process of increasing phage populations through replication within bacterial hosts. This natural process involves phages infecting bacterial cells, replicating, and producing numerous progenies of phages. The phage amplification assay (PAA) is used for detecting specific bacterial pathogens by leveraging phages that specifically infect the target bacteria [79]. The process starts by mixing the sample with phages, allowing them time to adsorb susceptible bacteria. Then, an antiviral agent (virucide) such as ferrous sulfate or tea extract is added to inactivate any extracellular phages, leaving only adsorbed particles, which subsequently engage in the infection process. To enhance detection, helper cells of a bacterial strain that supports phage growth are added to the sample after neutralizing the virucide (Figure 4) [79]. This assay can detect low levels of bacterial contamination because of phage amplification [80,81]. Capitalizing on the benefits of phage amplification assays, they have been combined with other methods such as spectrophotometry, quantitative PCR (qPCR), and loop-mediated isothermal amplification (LAMP) to detect viable *Salmonella* cells [21,58,65].

For example, Favrin et al. (2002) introduced an innovative immunomagnetic separation (IMS)-phage assay with four essential steps of capturing *Salmonella* cells using IMS, phage infection, recovery of phage progeny, and assessment of the phage progeny by evaluating their effects on a healthy population of host cells, referred to as signal amplifying cells (SACs) [65]. The assay detected an average of 3 CFU of *S*. Enteritidis per 25 g of skim milk powder, chicken meat, and ground beef samples.

Subsequently, phage amplification coupled with quantitative PCR (PAA-qPCR) enhanced the detection of *S*. Enteritidis in chicken meat samples [58]. After selectively infecting *Salmonella* cells with phage vB_SenS_PVP-SE2, progeny phages were produced and released into the sample. Following this, qPCR was used to detect nucleic acids of progeny phages rather than bacterial DNA. The findings revealed the detection of 0.22 fg/μL of pure viral DNA (equivalent to 10^3^ PFU/mL of phage particles). After a brief bacterial recovery phase, the introduction of phage into chicken samples spiked with 8 CFU/25 g resulted in detection within 10 h. In a similar experimental setup by another research group, PAA-qPCR was applied to detect *S*. Typhimurium in lettuce and skim milk [54]. Furthermore, utilizing phage T156, this method achieves a detection limit of 10 CFU/mL within 3.5 h, offering high specificity for live bacteria and excluding detection of dead bacteria. Additionally, the assay exhibits specific detection capabilities for drug- resistant *S*. Typhimurium, reducing the detection time from 24 h to 6.5 h, offering a significant time-saving advantage compared to traditional culture-based methods.

Incorporating a phage amplification assay with loop-mediated isothermal amplification (PA-LAMP), Lamas et al. (2023) detected viable *S*. Enteritidis via replicated phage DNA by using two strategies, a real-time fluorescence and naked-eye detection under UV light conditions [21]. The approach reached detection limits as low as 0.2 fg/μL of pure phage DNA (equivalent to 2.2 log PFU/mL). Both real-time fluorescence and naked-eye detection methods showed a limit of detection with 95% of confidence (LOD_95_) of 6.6 CFU/25 g and a limit of detection with 50% of confidence (LOD_50_) of 1.5 CFU/25 g in spiked chicken breast samples, completed within an 8 h timeframe. The PAA reaction conditions, including the infection concentration and time for each phage, need to be optimized for the most efficient amplification. For instance, using phage T156 at 10^5^ PFU/mL with a 30-min infection period yielded an optimal burst time for progeny phages within the same duration. Furthermore, ferrous ammonium sulfate (FAS) at a final concentration of 30 mM was stable and effectively neutralized all phages at 10^5^ PFU/mL within 8 min [54]. Under these optimized conditions, the PAA-qPCR method achieved a detection sensitivity of 1 CFU per 25 g sample of chicken meat or milk, with a total detection time of 3.5 h [54].

Overall, phage amplification assays provide a robust, efficient, and cost-effective method for the specific detection of bacterial pathogens in various settings, including food safety, clinical diagnostics, and environmental monitoring.

3. *Engineered reporter phages*

Reporter phages are modified to carry reporter genes, which allow for the detection of specific bacteria by producing easily measurable signals upon infection. Reporter genes can be chosen to produce various types of signals, such as luminescence (luciferase; *lux/luc*), fluorescence (*gfp*), or color change (beta-galactosidase; *lacZ*), thus making the detection process versatile and adaptable to different detection platforms [78]. The reporter phages can be used to detect foodborne pathogens like NTS in food products [23]. The rapid detection capability of the method can help in ensuring food safety and preventing outbreaks. The reporter *luciferase* gene is commonly used for detecting NTS in egg, iceberg lettuce, sliced pork, ground turkey, chicken breast, powdered infant formula, and milk [23,24,66].

The first luciferase reporter phage for the purpose of detecting NTS in food was constructed by Chen and Griffiths in 1996 [66]. Phage P22 was engineered to incorporate *lux* genes into its genome, enabling the detection of a luminescence signal using a photon-counting charge-coupled device (CCD) camera or X-ray film. This system was developed not only to detect *Salmonella* contamination in whole eggs but also to identify its precise location within the egg. It can detect 63 CFU/egg of *S*. Enteritidis contamination within 24 h.

Regarding phage life cycles, lysogenic reporter phages are more efficient for host detection than lytic phages, as the latter quickly destroy the host cell’s signal production machinery [82]. Temperate phage SPC32H was modified to include *luxCDABE*, generating a bioluminescent reporter signal [23]. From this study with the modified phage SPC32H-CDABE, at least 20 CFU/mL of pure *S*. Typhimurium was detectable within 2 h, with signal intensity that increased proportionally to cell contamination levels in lettuce, sliced pork, and milk. Also, unnecessary genes from the phage SPC32H genome, including the *int* integrase gene, were deleted, and it was found that the *int*-defective phage produced similar levels of bioluminescence. The phage demonstrated significant bioluminescence production without causing immediate lysis of the host *Salmonella*.

Further to the work on phage SPC32H, more luciferase reporter phages were engineered to express *NanoLuc*, an optimized *luciferase* derived from the deep-sea shrimp *Oplophorus gracilirostris*, which is 150 times brighter than traditional *luciferase* genes [24]. This development produced a more detectable bioluminescence signal and minimized background noise than the *luxCDABE* engineered phage. More studies from the same research group introduced an enhanced method for detecting NTS in food matrices using *NanoLuc*-expressing phages [24,55]. The combination of *NanoLuc*-expressing phages, SEA1 and TSP1, as a phage cocktail can accurately detect 1 CFU in either 25 g of ground turkey with a 7 h enrichment or 100 g of powdered infant formula with a 16 h enrichment [24]. In another study [55], strains of *S. enterica* were detected within 9 h in all lettuce samples containing 10 CFU or more per 25 g.

In summary, engineered reporter phages represent a promising and innovative approach for bacterial detection, offering high specificity, rapid results, and the ability to assess bacterial viability. Despite these advantages, their construction is labor-intensive and demands a thorough understanding of phage genetics. The limited volume of the phage capsid poses constraints on genetic material incorporation. Techniques such as direct cloning, transposition, and homologous recombination are employed for gene introduction, albeit applicable to only certain phages. Additionally, their use involves genetically modifying host cells, which raises concerns regarding consumer acceptance and regulatory approval.

4. *Phages as bio-probes or phage biosensors*

Phages are increasingly utilized as bio-probes or biosensors due to their unique ability to specifically recognize and interact with bacterial targets. Phage biosensors, a notable advancement in phage-based bacterial detection, illustrate this trend. These biosensor platforms typically involve the use of either whole phages or partial phage particles. Upon infecting the host bacterium, they produce detectable signals such as colorimetric, electrical, fluorescent, or luminescent signals [83]. The use of phages as bio-probes to detect NTS in milk was developed by Hirsh and Martin in 1983, who aimed to minimize cross-reactivity associated with immunological agents such as antibodies [67]. The scheme employs the *Salmonella*-specific phage Felix-O1, which proliferates in the presence of NTS. The increased number of phages is detected using high-pressure liquid chromatography (HPLC), successfully identifying the presence of fewer than five *Salmonella* per milliliter of milk within 24 h.

Defined as a simple but novel detection concept, a demonstration involved combining an affinity capture surface with an intracellular metabolic marker to visually detect the presence of bacteria on the affinity surface using colorimetry. This method can detect as little as 5 CFU/25 g of *S*. Typhimurium and *S*. Napoli, unaffected by the presence of food particles and the natural background microflora [62]. Phage-based magnetoelastic (ME) biosensors were utilized to directly detect *S. enterica* in foods. The detection signal originates from ME materials, which are magnetostrictive alloys that change their magnetic properties in response to mechanical stress and vice versa. In this scenario, the binding of bacterial cells to the phage coated ME material increases the sensor’s mass. This mass loading alters the mechanical vibration characteristics of the ME material and the biosensor platform successfully detected *S*. Typhimurium at concentrations of 500 CFU/mL in spiked fresh tomato [63], 100 CFU/25 g on spinach leaves [60], and 1.31 to 1.85 log CFU/mL on *Salmonella*-inoculated chili peppers [48].

The surface-scanning ME biosensor was tested on modified filamentous E2 phages, immobilized on the sensor, establishing distinct resonant frequencies and making them useful for *Salmonella* detection in food matrices [84]. However, a study has shown that the wild tailed phage (TST) demonstrates greater sensitivity and binding affinity than the modified filamentous E2 phage, achieving a lower detection limit of detection (LOD) of 1.31 ± 0.27 log CFU/mL of *S*. Typhimurium tested on chili peppers, whereas the LOD value found in E2 phages was lower, at 1.85 ± 0.31 log CFU/mL [48].

Phage biosensors for rapid detection of NTS (total detection time not exceeding 2 h) were implemented across various platforms. Five studies were developed to detect targeted bacteria within 30 min, without requiring an enrichment step. Firstly, a phage F5-4 (capture probe)-based lateral flow immunoassay (LFIA) coupled with surface- enhanced Raman spectroscopy (SERS) achieves the lowest LOD of 7 CFU/mL for *S*. Enteritidis in chicken meat and egg samples [57]. Secondly, S. Wang et al. (2023) reported a phage/DNAzyme co-modified zeolitic imidazolate framework-encoded bio-probe that detected *S*. Typhimurium in spiked milk, pork, fish, and lettuce samples, achieving a limit of detection (LOD) of 8 CFU/mL [49]. This strategy showcases the feasibility of *S*. Typhimurium, enabling on-site detection of contamination levels in foods. Thirdly, a novel electrochemical biosensor was introduced. The biosensor is constructed by electrostatically immobilizing phage vB_SenM_PA13076 onto MXene-nanostructured electrodes. MXene, known for its high surface area, biocompatibility, and conductivity, enables dense phage immobilization while maintaining phages’ viability and functionality for pathogen detection. The biosensor demonstrates enhanced sensitivity, with a low LOD of 5 CFU/mL, and excellent specificity, even in the presence of other foodborne bacteria [37]. Fourthly, the ultrasensitive electrochemical detection of *S*. Typhimurium in food matrices was reported. The biosensor uses surface-modified bacterial cellulose (BC) integrated with polypyrrole (Ppy) and reduced graphene oxide (RGO), functionalized with immobilized *S*. typhimurium-specific phage particles. The BC substrate’s fibrous and porous structure is enhanced by the oxidative polymerization of Ppy and RGO, creating a conductive and flexible bio-interface. It provided detection limits of 1 CFU/mL in PBS, 5 CFU/mL in milk, and 3 CFU/mL in chicken meat [38]. Finally, in rapid detection within 20 min, the Det7 phage tail protein, used with a surface plasmon resonance (SPR) biosensor, enabled detection of *S*. Typhimurium at 5 × 10^4^ CFU/mL in contaminated apple juice [56]. A relatively low sensitivity appears to be a common limitation of using SPR biosensors for bacterial detection, as demonstrated in previous studies [85,86].

Utilizing a detection time of approximately 2 h, a bioorthogonal reaction-mediated particle-counting sensing platform [53] was developed. Bioorthogonal reactions are high-yielding chemical reactions that can occur in organisms or simulated microenvironments using low-concentration, non-toxic reagents such as tetrazine ligation, the cycloaddition of s-tetrazine (Tz) with *trans*-cyclooctene dienophiles (TCO) [87]. This platform was used for signal amplification in a phage LPST10-mediated sensor, enabling rapid detection of *S*. Typhimurium within 1 h in food matrices [53]. Incorporating UV-spectrophotometry [45] and quantum dot fluorescence phage biosensors [46] achieved detection time frames of 80 min and 2 h, respectively. According to Anany et al. (2018), the first bioactive phage-based paper dipstick, when combined with qPCR, detects *S*. Newport in chicken homogenate samples with a detection limit of 50 CFU/mL [59]. To achieve the lowest LOD of 1 CFU/mL, a phage-based electrochemical impedance spectroscopy biosensor was coupled with a 3.5-h pre-enrichment process in chicken breast samples [52].

In contrast to other studies, Muldoon et al. (2007) proposed the idea that using specific phage cocktails to control non-*Salmonella* species in food metrices such as *Citrobacter* spp. and *E. coli* could reduce false-positive results [64]. After removing non-target bacteria, serogroup-specific monoclonal antibodies on an immunochromatographic strip were used to detect NTS in raw ground beef, liquid egg, and sliced cooked turkey [64]. When compared to the FSIS-USDA cultural reference method, the developed method demonstrated 100% sensitivity and specificity in detecting NTS in contaminated raw ground chicken [64]. Based on the evidence provided, phage-based biosensors serve as a robust and adaptable tool for detecting bacterial contaminants in food, delivering rapid, sensitive, and specific results vital for safeguarding food safety and public health. Ongoing research endeavors seek to enhance these technologies by improving detection limits, accelerating assay times, and ensuring compatibility across diverse food matrices, thereby expanding their utility in food safety and beyond.

## 4. Discussion

Phage-based detection methods offer notable advantages over traditional approaches, particularly in their specificity and efficacy against pathogenic bacteria. Phages exhibit high selectivity for viable bacterial hosts or specific strains, ensuring that detection systems accurately target active *Salmonella* infection or contamination while minimizing cross-reactivity with other microorganisms [88]. Whole phages are typically suitable for this purpose due to their innate ability to target and infect particular bacterial strains, but they can also be genetically engineered to enhance specificity. For example, the modifications of tail fiber proteins, which play a critical role in host recognition and to initiate infection, have been shown to improve phage target capacity and to differentiate between closely related bacterial strains, thereby increasing the accuracy of detection systems [20,89].

Phage selectivity is essential for applications in complex food matrices and environments with diverse microbial communities, where the presence of non-target bacteria can potentially complicate detection efforts [90]. However, dairy matrices such as milk and cheese are characterized by high concentrations of proteins and fats, which can significantly impede phage–host interactions [91]. Similarly, the detection of foodborne pathogens in meat is also challenged by the presence of blood components (in addition to fats and proteins) and in fruit juices with a low pH, which are known to impact the activity of specific phages [91]. Despite these limitations, phage detection methods for NTS have been successfully applied to various food types. These food types include dairy products such as milk, as well as meat products such as chicken, and fresh produce including fruits and vegetables [26]. To perform this effectively and to ensure optimal phage activity in these complex food matrices, rigorous sample preparation protocols encompassing filtration, dilution, centrifugation, and enzymatic treatments are imperative to eliminate inhibitory substances and mitigate matrix-associated interferences [91].

Further, to sample pre-treatment protocols, the initial phase of phage-based detection entails the recovery or enrichment of *Salmonella* from the food samples. The sensitivity of these methods was shown to be significantly improved by optimizing both the concentration of whole phages or phage-derived proteins and the conditions under which they interact with the bacterial cells [26]. This combined strategy can enhance the effectiveness of traditional culture-based methods by reducing the time required and minimal *Salmonella* benchmark concentration for robust identification [12]. For example, typical *Salmonella* detection techniques using qPCR produced results within fewer hours when combined with phage enrichment than when used alone, which is essential for timely intervention in food safety management [58]. This strategy further supports the seamless integration of phage-based detection methods with other conventional techniques, such as colony counting on selective agar, enzyme-linked immunoassays (ELISA), and real-time PCR, to enhance detection. Additionally, LTF4-a conjugated with MNPs has been shown to enhance recovery efficiency [51]. This integration allows for a more comprehensive and confirmatory approach to NTS detection [92]. In addition to combined detection systems, parameters such as reaction volume, incubation time, pH, and NaCl concentration play critical roles in maximizing the binding efficiency and recovery of *Salmonella* cells.

While the advantages of phage-based NTS detection methods are compelling, several practical considerations and challenges must be addressed before widespread implementation. One of the primary challenges is the regulatory landscape. Phage detection methods are relatively novel, and existing regulatory frameworks may not fully address their use. Gaining approval for these methods requires demonstrating their safety, efficacy, reliability and reproducibility, which can vary depending on the specific phage strains used [76]. Each phage detection system may need individual evaluation for regulatory approval, adding complexity to the process, especially when different phage cocktails are involved.

Another significant challenge is public understanding and acceptance of phage-based technologies in food safety. Misconceptions or lack of awareness about the safety and benefits of the detection could hinder or delay adoption [93]. Although there is a growing interest in developing phage-based detection methods, most of these technologies remain at the experimental stage in laboratories, and their translation to commercial products has been grossly slow. Transparent communication and education efforts are necessary to build consumer trust.

Another key limitation of phage-based bacterial detection is the narrow host range of phages, which can lead to missed detections, particularly with strain/serovar diversity in NTS [94]. To address this, a cocktail of phages may be needed, but this can complicate the assay and increase the risk of phage interference [95]. Rather than covering all serovars of NTS, priority should be given to the development of detection methods for the two most significant *Salmonella* serovars transmitted from animals to humans worldwide [2].

Just as bacteria can develop resistance to phage-based therapies, there is a risk that resistance could also develop in the context of detection, potentially compromising the effectiveness of the system over time [96]. This necessitates ongoing monitoring and possible adjustments to the phage selection process. Additionally, the widespread use of phages in detection systems raises concerns about their potential impact on natural microbial communities if released into the environment [97]. Careful assessment and management of these ecological risks are essential to prevent unintended consequences.

Addressing these challenges requires a collaborative approach involving scientists, regulatory bodies, industry stakeholders, and public health experts to develop, validate, and implement phage-based detection systems that are effective, safe, and economically viable.

## 5. Conclusions

Phage-based detection methods for NTS in foods demonstrate high specificity, sensitivity, and rapid detection capabilities. By optimizing recovery and enrichment steps and combining phage-based techniques with other detection methods, these approaches can enhance the overall efficiency of NTS detection in food safety applications. Further research and development are essential to address the challenges and fully realize the potential of phage-based detection systems for NTS to ensure food safety. The successful development of a highly effective phage-based NTS detection system can serve as a model for implementation in the detection of other foodborne pathogens.

## Figures and Tables

**Table 1:** The efficacy of phages or phage components for detecting non-typhoidal *Salmonella* in various food samples.

*S. enterica* Serovar (s)	Types of Food Sample	Phage Identification	Phage Family	Phage/Phage Components	Host Range of Phage/Phage Components	Pre-Enrichment Time	Detection Method	Limit of Detection (LOD)	Total Detection Time	Ref.
*S*. Enteritidis	Eggs, milk	vB_SenM_PA13076	*Myoviridae*	Whole particle	Not reported	Not required	Electrochemical impedance spectroscopy (EIS)	5 CFU/mL	30 min	[37]
*S*. Typhimurium	Chicken meat	*S*. Typhimurium-specific phage	*Siphoviridae*	Whole particle	Not reported	Not required	Electrochemical impedance spectroscopy (EIS)	5 CFU/mL	30 min	[38]
	Milk							3 CFU/mL		
*S*. Derby, *S*. London, *S*. Anatum, *S.* Choleraesuis, *S*. Meleagridis, *S*. Typhimurium, *S.* Thompson, *S*. Rissen, *S*. Kottbus, *S.* Litchfield, *S*. Enteritidis, *S*. Singapore	Milk	Felix O-1	*Myoviridae*	Whole particle	*S*. Derby (n = 1), *S*. London (n = 1), *S*. Anatum (n = 1), *S*. Choleraesuis (n = 1), *S.* Meleagridis (n = 1), *S*. Typhimurium (n = 2), *S.* Thompson (n = 1), *S*. Rissen (n = 1), *S*. Kottbus (n = 1), *S*. Litchfield (n = 1), *S*. Enteritidis (n = 3), *S*. Singapore (n = 1)	Required (3 h)	Magnetic separation combined with real-time recombinase polymerase amplification	10 CFU/25 mL	3.5 h	[39]
*S*. Typhimurium	Pork, milk	S.T 15, S.T 16	Not reported	Whole particle	Not reported	Not required	Magnetic separation combined with bioluminence and colorimetric assay	30 CFU/mL	40 min	[40]
*S*. Typhimurium	Pork, fish, milk	Phage 1 (KM15), Phage 2 (KM16)	*Myoviridae*	Whole particle	*S*. Typhimurium (n = 3), *S*. Choleraesuis (n = 1), *S*. Enteritidis (n = 1), *S. bongori* (n = 1)	Not required	Bioluminence and pressure assay	30 CFU/mL for live cells 40 CFU/mL for total cells	30 min	[41]
*S*. Typhimurium	Chicken meat	LPST10	*Myoviridae*	Whole particle	*S*. Typhimurium (n = 3), *S*. Enteritidis (n = 3), *S*. Paratyphi B (n = 1)	Not required	Magnetic separation combined with fluorescence	20 CFU/mL	1.5 h	[42]
*S*. Typhimurium	Pork, Milk	P22	*Podoviridae*	Tail spike protein	*S*. Typhimurium	Not required	Magnetic separation combined with colorimetric smartphone-assisted paper sensor	4620 CFU/mL	2.5 h	[43]
*S*. Enteritidis	Milk	LPSE1	*Siphoviridae*	Whole particle	*S*. Enteritidis (n = 3), *S*. Typhimurium (n = 2), *S*. Choleraesuis (n = 1), *S*. Paratyphi B (n = 1),	Required (50 min)	Magnetic separation combined with fluorescence	6 CFU/mL	2 h	[44]
*S.* Typhimurium	Lettuce	T156	*Siphoviridae*	Whole particle	*S*. Typhimurium (n = 13), *S*. Enteritidis (n = 12), *S*. Agona (n = 3), *S*. Indiana (n = 2) *S*. Paratyphi B (n = 1), *S*. Choleraesuis (n = 1), *S*. Gallinarum (n = 2), *S*. Dublin (n = 2)	Not required	UV-spectrophotometry	38 CFU/mL	80 min	[45]
*S*. Pullorum, *S*. Dublin, *S*. Enteritidis, *S*. Typhimurium, *S*. Agona, *S*. Indiana	Lettuce skim milk	STP55	*Ackermannviridae*	Phage receptor binding protein	*S*. Enteritidis (n = 10), *S*. Typhimurium (n = 12), *S*. Agora (n = 2), *S*. Indiana (n = 2), *S*. Paratyphi B (n = 1), *S*. Pullorum (n = 2), *S*. Dublin (n = 2), *E. coli* (n = 2), *E. coli* O157:H7 (n = 1)	Not required	Quantum dot fluorescence	2 CFU/mL	2 h	[46]
*S*. Anatum, *S*. Choleraesuis, *S*. Enteritidis, *S*. Dublin *S*. Typhimurium, *S*. Kentucky, *S*. Newport, *S*. Javiana, *S*. Arizonae	Chicken breast, lettuce, Skim milk	T102	*Siphoviridae*	Phage receptor binding protein	*S*. Typhimurium (n = 11), *S*. Enteritidis (n = 8), *S*. Pullorum (n = 1), *S*. Javiana (n = 1), *S*. Dublin (n = 2), *S*. Agona (n = 1)	Not required	Magnetic microbead enzyme-linked immune- assay (MELISA)	10 CFU/mL	1.5 h	[47]
*S*. Typhimurium	Chili pepper	TST, FST	*Inoviridae*	Whole particle	Not reported	Not required	Magnetoelastic (ME)	1.31 ± 0.27 log CFU/mL (TST) 1.85 ± 0.22 log CFU/mL (FST)	1 h	[48]
*S*. Typhimurium	Milk, pork, fish, lettuce	Not reported	Not reported	Whole particle	*S*. Typhimurium	Not required	Fluorescence and electrochemistry	8 CFU/mL	30 min	[49]
*S*. Enteritidis	Chicken breast	vB SenS_PVP-SE2	*Siphoviridae*	Whole particle	*S*. Enteritidis	Required (3 h)	Loop-mediated isothermal amplification (LAMP)	6.6 CFU/25 g	8 h	[21]
*S*. Typhimurium, *S*. Enteritidis	Orange juice	LPST10	*Siphoviridae*	Whole particle	*S*. Typhimurium (n = 3), *S*. Enteritidis (n = 3), *S*. Paratyphi B (n = 1)	Required (4 h)	Magnetic relaxation switching (MRS)	Less than 10 CFU/25 mL	4.5 h	[50]
*S*. Typhimurium	Egg, lettuce, fresh milk	STP4-a	*Myoviridae*	Long tail fiber protein	*S*. Enteritidis (n = 29), *S*. Typhimurium (n = 14), *S*. Chloreasuis (n = 3), *S*. Senftenberg (n = 2) *S*. Newport (n = 3), *S*. Poona (n = 2), *S*. Tenessee (n = 2), *S*. Kentucky (n = 1), *S*. Anatum (n = 1), *S*. Dublin (n = 1), *S*. St. Paul (n = 1), *S*. Schwarzengrund (n = 1), *S*. Montevideo (n = 3), *S*. Cerro (n = 1), *S*. Infantis (n = 4), *S*. Winston (n = 2), *S*. Minnesota (n = 1), *S*. Amager (n = 1), *S*. Orion (n = 1), *S*. Rissen (n = 1), *S*. Derby (n = 1), *S*. Dabou or Corvallis (n = 1), *S*. Molade (n = 1), *Salmonella* spp. (n = 11)	Not required	Magnetic separation combined with real-time PCR	7 CFU/mL	3 h	[51]
*S*. Enteritidis	Chicken breast, lettuce	SEP37	*Myoviridae*	Whole particle	*S*. Typhimurium (n = 14), *S*. Enteritidis (n = 9), *S*. Paratyphi B (n = 1), *S*. Pullorum (n = 2), *S*. Duck (n = 1), *S*. Choleraesuis (n = 1), *S*. Dublin (n = 2), *S*. Agona (n = 2), *S*. Indiana (n = 2)	Required (3.5 h)	Electrochemical impedance spectroscopy (EIS)	1 CFU/mL	4 h	[52]
*S*. Typhimurium	Chicken meat milk, orange juice	LPST10	*Siphoviridae*	Whole particle	*S*. Typhimurium (n = 3), *S*. Enteritidis (n = 3), *S*. Paratyphi B (n = 1)	Required (1 h)	Microparticle counting sensing	33.58 CFU/mL	1 h	[53]
*S*. Typhimurium	Lettuce, Milk	T156	*Siphoviridae*	Whole particle	*S*. Typhimurium (n = 13), *S*. Enteritidis (n = 12), *S*. Agona (n = 3), *S*. Indiana (n = 2) *S*. Paratyphi B (n = 1), *S*. Choleraesuis (n = 1), *S*. Gallinarum (n = 2), *S*. Dublin (n = 2)	Not required	Real-time PCR	1 CFU/mL	3.5 h	[54]
*S*. Minnesota, *S*. Enteritidis, *S*. Saintpaul, *S*. Infantis, *S*. Heidelberg, *S*. Typhimurium	Lettuce	SEA1, TSP2	*Myoviridae*	Whole particle	Not reported	Required (7 h)	Luminescence	10 CFU/25 g or more	9 h	[55]
*S*. Typhimurium	Apple juice	Det7T	*Myoviridae*	Tail protein	Not reported	Not required	Surface plasmon resonance (SPR)	50,000 CFU/mL	20 min	[56]
*S*. Enteritidis	Chicken meat, egg	F5–4	Not reported	Whole particle	Not reported	Not required	Lateral flow immunoassay (LFIA) combined with Surface-enhanced Raman spectroscopy (SERS)	7 CFU/mL	30 min	[57]
*S*. Newport	Ground turkey	SEA1, TSP1	*Myoviridae*	Whole particle	Not reported	Required (7 h)	Luminescence	1 CFU/25 g	9 h	[24]
*S*. Muenter	Powdered infant formula					Required (16 h)		1 CFU/100 g	18 h	
*S*. Enteritidis	Chicken breast	vB SenS_PVP-SE2	*Siphoviridae*	Whole particle	*S*. Enteritidis	Required (3 h)	Real-time PCR	8 CFU/25 g	10 h	[58]
*S*. Newport	Chicken meat	vB_SnwM CGG4-1	*Myoviridae*	Whole particle	Not reported	Not required	Phage printed onto paper strips using modified inkjet combined with real-time PCR	50 CFU/mL	8 h	[59]
*S*. Typhimurium	Fresh spinach leaves	E2	*Inoviridae*	Whole particle	*S*. Typhimurium	Required (7.5 h)	Magnetoelastic (ME) biosensors	100 CFU/25 g	7.5 h	[60]
*S*. Typhimurium	Chicken meat, infant formula, milk, chocolate milk	S16	*Myoviridae*	Long tail fiber protein	*S*. Typhimurium (n = 7), *S*. Enteritidis (n = 1), *S*. Hadar (n = 1), *S*. Heidelberg (n = 1), *S*. Indiana (n = 1), *S*. Infantis (n = 1), *S*. Javiana (n = 1), *S*. Montevideo (n = 1), *S*. Newport (n = 2), *S*. *enterica* subsp. *arizonae* (n= 1), *S*. *enterica* subsp. *diarizonae* (n = 1), S. *enterica* subsp. *salamae* (n = 1), S. *enterica* subsp. *houtenae * (n = 1), *S*. *bongori* (n = 1)	Not required	Enzyme-linked LTF assay and colony counting on XLD agar	1 to 10 CFU/25 g or mL	1 h	[22]
	Celery, alfalfa sprouts					Required (overnight)			24 h	
*S*. Typhimurium	Milk	P22	*Siphoviridae*	Whole particle	Not reported	Required (6 h)	Magnetic separation combined with colorimetric immunoassay	19 CFU/mL	2.5 h	[61]
*S*. Typhimurium	Iceberg lettuce	SPC32H	*Podoviridae*	Whole particle	Not reported	Not required	Luminescence	22 ± 10.4 CFU/g	2 h	[23]
	Sliced pork							37 ± 10.4 CFU/g		
	Milk							700 ± 11.6 CFU/mL		
*S.* Typhimurium, *S.* Napoli	Ground beef, egg, pastry	Not reported	Not reported	Tail protein	Not reported	Not required	Magnetic separation combined with colorimetry	5 CFU/25 g	22 h	[62]
*S*. Typhimurium	Fresh tomato	E2	*Inoviridae*	Whole particle	*S*. Typhimurium	Not required	Magnetoelastic (ME)	500 CFU/mL	45 min	[63]
*S*. Typhimurium	Raw ground beef	Not reported	Not reported	Whole particle	Not reported	Required (16 to 22 h)	Immunochromatography	0.092 most probable number (MPN)/g	6 to 22 h	[64]
*S*. Enteritidis	Liquid egg					Required (6 to 8 h)		0.043 most probable number (MPN)/g		
*S*. Kentucky	Sliced cooked turkey					Required (6 to 8 h)		0.15 most probable number (MPN)/g		
*S.* Enteritidis	Skim milk powder, chicken meat, ground beef	vB_SalM_SJ2	*Ackermannviridae*	Whole particle	Not reported	Required (16 h)	Immunomagnetic separation (IMS) combined with spectrophotometry (optical density; OD)	3 CFU/25 g or mL	20 h	[65]
*S*. Enteritidis	Egg	P22	*Siphoviridae*	Whole particle	Not reported	Not required	Luminescence	63 CFU/ Egg	24 h	[66]
*S*. Dublin, *S*. Typhimurium, *S*. Anatum, *S*. Krefeld, *S*. Saintpaul	Milk	Felix-O1	*Myoviridae*	Whole particle	*S*. Derby (n = 1), *S*. London (n = 1), *S*. Anatum (n = 1), *S*. Choleraesuis (n = 1), *S*. Meleagridis (n = 1), *S*. Typhimurium (n = 2), *S*. Thompson (n = 1), *S*. Rissen (n = 1), *S*. Kottbus (n = 1), *S*. Litchfield (n = 1), *S*. Enteritidis (n = 3), *S*. Singapore (n = 1)	Required (overnight)	Liquid chromatography	Less than 5 CFU/mL	24 h	[67]

**Table 2:** The assessed methodological quality of the 35 individual studies using the JBI Critical Appraisal Checklist for Systematic Reviews.

Title	Details of Study		Methodological Quality
Bacterial Enrichment	Country (Year)	%Recovery Efficiency ± RSD	
Phage-based magnetic capture method as an aid for real-time recombinase polymerase amplification detection of *Salmonella* spp. in milk	China (2024)	Ranging from 65.00 to 90.00	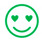
A microfluidic chip platform based on Pt nanozyme and magnetized phage composite probes for dual-mode detecting and imaging pathogenic bacteria viability	China (2024)	Ranging from 81.20 to 98.90 ± 2.10 to 7.50 (Live cells) Ranging from 89.00 to 102.00 ± 2.30 to 8.40 (Total cells)	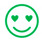
Specific separation and sensitive detection of foodborne pathogens by phage-derived bacterial-binding protein-nano magnetic beads coupled with smartphone-assisted paper sensor	China (2024)	92.00 ± 5.82	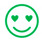
Magnetic microbead enzyme-linked immunoassay based on phage encoded protein RBP 41-mediated for rapid and sensitive detection of *Salmonella* in food matrices	China (2023)	83.43 ± 2.15	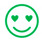
A phage-based magnetic relaxation switching biosensor using bioorthogonal reaction signal amplification for *Salmonella* detection in foods	China (2023)	94.30 ± 8.30	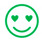
Phage long tail fiber protein-immobilized magnetic nanoparticles for rapid and ultrasensitive detection of *Salmonella*	China (2022)	98.00 ± 2.00	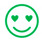
Modified bacteriophage S16 long tail fiber proteins for rapid and specific immobilization and detection of *Salmonella* cells	Switzerland (2017)	98.00 ± 1.00	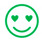
Phagomagnetic immunoassay for the rapid detection of *Salmonella*	Spain (2014)	Not reported	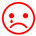
**Bacterial Detection**	**Country (Year)**	**Significant Results**	**Methodological Quality**
An electrochemical biosensor for sensitive detection of live *Salmonella* in food via MXene amplified methylene blue signals and electrostatic immobilization of Bacteriophages	China (2024)	Recovery rates: 98.30% to 100.30% (milk) 99.20% to 102.20% (eggs) R^2^ = 0.9958	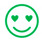
Ultrasensitive electrochemical detection of *Salmonella* Typhimurium in food matrices using surface-modified bacterial cellulose with immobilized phage particles	China (2024)	Linear range of detection = 10^0^–10^6^ CFU/mL R^2^ = 0.993 (milk) R^2^ = 0.974 (chicken meat)	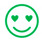
Versatile platinum nanoparticles-decorated phage nanozyme integrating recognition, bacteriolysis, and catalysis capabilities for on-site detection of foodborne pathogenic strains vitality based on bioluminescence/pressure dual-mode bioassay	China (2024)	Linear range of detection = 10^2^–10^7^ CFU/mL R^2^ = 0.991	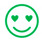
DNA and ATP synergistically triggered Argonaute-mediated sensor for the ultrasensitive detection of viable *Salmonella* without DNA extraction and amplification	China (2024)	Recovery rates: 87.72% to 113.97% with 3.72–6.88% of CVs	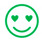
A caprylate esterase-activated fluorescent probe for sensitive and selective detection of *Salmonella* Enteritidis	China (2023)	Specificity = 100% Sensitivity = Not reported (n = 8)	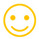
A visual colorimetric assay based on phage T156 and gold nanoparticles for the sensitive detection of *Salmonella* in lettuce	China (2023)	Recovery rates: 81.00% to 119.20% with 3.3–14.7% of RSDs	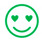
Quantum dot-labeled phage-encoded RBP 55 as a fluorescent nanoprobe for sensitive and specific detection of *Salmonella* in food matrices	China (2023)	Recovery rates = 90.00–110.00% with 2 below 9.50% of CVs	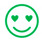
Performance of wild, tailed, humidity- robust phage on a surface-scanning magnetoelastic biosensor for *Salmonella* Typhimurium detection	South Korea (2023)	Sensitivities: Phage TST = 880.80 ± 11.31 Phage FST = 808.60 ± 24.11 Hz/(log CFU/mL)]	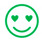
Tesla valve-assisted biosensor for dual-mode and dual-target simultaneous determination of foodborne pathogens based on phage/DNAzyme co-modified zeolitic imidazolate framework-encoded probes	China (2023)	Recovery rates = 90.00–110.00% with 3.55–11.10% of RSDs	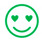
Phage amplification coupled with loop- mediated isothermal amplification (PA-LAMP) for same-day detection of viable *Salmonella* Enteritidis in raw poultry meat	Spain (2023)	Relative specificity = 100% Relative sensitivity = 100% Relative accuracy = 100% (n = 29)	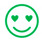
EIS biosensor based on a novel *Myoviridae* bacteriophage SEP37 for rapid and specific detection of *Salmonella* in food matrixes	China (2022)	Recovery rates = 94.00–105.00%	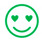
Novel bioorthogonal reaction-mediated particle counting sensing platform using phage for rapid detection of *Salmonella*	China (2022)	Recovery rates = 85.58–99.03% with 2.56–8.37% of CVs	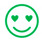
Phage amplification-based technologies for simultaneous quantification of viable *Salmonella* in foodstuff and rapid antibiotic susceptibility testing	China (2022)	R^2^ = 0.9985 (milk) R^2^ = 0.9496 (lettuce) (Compared to conventional plate counting)	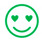
Evaluation of phageDX *Salmonella* assay for *Salmonella* detection in hydroponic curly lettuce	Brazil (2021)	LOD = 10 CFU/25 g or more	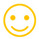
Novel surface plasmon resonance biosensor that uses full-length Det7 phage tail protein for rapid and selective detection of *Salmonella enterica* serovar Typhimurium	South Korea (2021)	LOD = 5.0 × 10^4^ CFU/mL	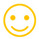
Replacement of antibodies with bacteriophages in lateral flow assay of *Salmonella* Enteritidis	Turkey (2021)	LOD = 7 CFU/mL	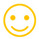
Accurate and sensitive detection of *Salmonella* in foods by engineered bacteriophages	USA (2020)	Specificity = 100% Sensitivity = 100% (n = 30)	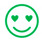
Specific detection of viable *Salmonella* Enteritidis by phage amplification combined with qPCR (PAA-qPCR) in spiked chicken meat samples	Portugal (2019)	Relative specificity = 100% Relative sensitivity = 96.60% Relative accuracy = 97.60% (n = 10)	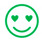
Print to detect: a rapid and ultrasensitive phage-based dipstick assay for foodborne pathogens	Canada (2018)	LOD = 50 CFU/mL	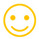
Detection of *Salmonella* Typhimurium on spinach using phage-based magnetoelastic biosensors	USA (2017)	LOD = 100 CFU/25 g	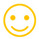
Development of an engineered bioluminescent reporter phage for the sensitive detection of viable *Salmonella* Typhimurium	South Korea (2014)	LOD = 20 to 700 CFU/g or mL	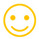
Simplified detection of food-borne pathogens: an in situ high affinity capture and staining concept	France (2012)	LOD = 5 CFU/25 g	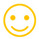
Direct detection of *Salmonella* Typhimurium on fresh produce using phage-based magnetoelastic biosensors	China (2010)	LOD = 500 CFU/mL	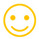
Bacteriophage-based enrichment coupled to immunochromatographic strip-based detection for the determination of *Salmonella* in meat and poultry	USA (2007)	Specificity = 100% Sensitivity = 100% (n = 60)	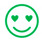
Application of a novel immunomagnetic separation bacteriophage assay for the detection of *Salmonella* Enteritidis and *Escherichia coli* O157:H7 in food	Canada (2003)	LOD = 3 CFU/25 g or mL	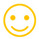
*Salmonella* detection in eggs using LuX (+) bacteriophages	Canada (1996)	LOD = 63 CFU/ Egg	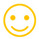
Detection of *Salmonella* spp. in milk by using Felix-O1 bacteriophage and high-pressure liquid chromatography	USA (1983)	LOD = Less than 5 CFU/mL	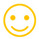

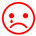
 = low methodological quality; 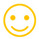 = moderate methodological quality; 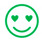 = high methodological quality. CV, coefficient of variation; LOD, limit of detection; R^2^; coefficient of determination*;* RSD, recovery and relative standard deviation.

## Data Availability

The original contributions presented in the study are included in the article/Supplementary Material. Further inquiries can be directed to the corresponding author.

## Supplementary Materials

The following supporting information can be downloaded at: https://www.mdpi.com/article/10.3390/v16121840/s1, Table S1: Search strategy in PubMed (Search date: 4 November 2024); Table S2: Search strategy in Embase (Search date: 4 November 2024); Table S3: Search strategy in ScienceDirect (Search date: 4 November 2024); Table S4: Quality assessment of the individual included studies (n = 35) using the JBI Critical Appraisal Checklist for Systematic Reviews. The methodological quality of each study was categorized as high, moderate, or low based on the evaluation of key critical domains for each checklist item.
