# Supplementary material for: Efficiency of Bacteriophage-Based Detection Methods for Non-Typhoidal Salmonella in Foods: A Systematic Review
Source: Viruses. 2024 Nov 27;16(12):1840. doi: 10.3390/v16121840 (PMC11680155; doi:10.3390/v16121840)
Supplement: Supplementary file 1 [file viruses-16-01840-s001.zip › viruses-3286429-supplementary.pdf]

Table S1. Search strategy in PubMed (Search date: November 4, 2024)

Step	Searching query	Results
#1	"bacteriophages"[tw]	31,683
#2	"phages"[tw]	23,507
#3	"Salmonella"[tw]	105,868
#4	(("bacteriophages"[tw]) OR ("phages"[tw])) AND ("Salmonella"[tw])	3,500
#5	"detection"[tw]	1,237,417
#6	((("bacteriophages"[tw]) OR ("phages"[tw])) AND ("Salmonella"[tw])) AND ("detection"[tw])	197

Table S2. Search strategy in Embase (Search date: November 4, 2024)

Step	Searching query	Results
#1	bacteriophages*:ti,ab,kw,de	13,941
#2	phages*:ti,ab,kw,de	18,069
#3	Salmonella*:ti,ab,kw,de	131,262
#4	#1 AND #2 AND #3	516
#5	detection*:ti,ab,kw,de	1,690,856
#6	#4 AND #5	73

Table S3. Search strategy in ScienceDirect (Search date: November 4, 2024)

Step	Searching query	Results
#1	" Salmonella bacteriophages"	263
#2	"Salmonella phages"	1,098
#3	"Salmonella bacteriophages" OR "Salmonella phages"	1,272
#4	"Salmonella bacteriophages" OR "Salmonella phages" AND "detection"	652
#5	"Salmonella bacteriophages" OR "Salmonella phages" AND "detection" AND "foods"	469
#6	"Salmonella bacteriophages" OR "Salmonella phages" AND "detection" AND "clinical"	417
#7	"Salmonella bacteriophages" OR "Salmonella phages" AND "detection" AND "foods" AND "clinical"	337

Table S4. Quality assessment of the individual included studies (n = 35) using the JBI Critical Appraisal Checklist for Systematic Reviews. The methodological quality of each study was categorized as high, moderate, or low based on the evaluation of key critical domains for each checklist item.

Q1*: Is the review question clearly and explicitly stated?
Q2*: Were the inclusion criteria appropriate for addressing the review question?
Q3*: Did the study provide fully available details of data?
Q4*: Was the measurement of the intervention conducted with validity and reliability?
Q5: Were the criteria for appraising studies appropriate?
Q6: Was critical appraisal conducted by two or more reviewers independently?
Q7: Were there methods to minimize errors in data extraction?
Q8: Were the methods used to combine studies appropriate?
Q9: Was the likelihood of publication bias assessed?
Q10: Were recommendations for policy and/or practice supported by the reported data?
Q11: Were the specific directions for future research appropriate?

* indicates critical question

**No.**	**Studies**	**Questions**											**Overall appraisal**
		**Q1***	**Q2***	**Q3***	**Q4***	**Q5**	**Q6**	**Q7**	**Q8**	**Q9**	**Q10**	**Q11**	
**Bacterial enrichment**													
1	J. Liu et al., 2024	Y	Y	Y	Y	Y	Y	Y	Y	N	N	Y	Hight
2	B. Liu et al.,2024	Y	Y	Y	Y	Y	Y	Y	Y	N	N	Y	Hight
3	Hong et al., 2024	Y	Y	Y	Y	Y	Y	Y	Y	N	N	U	High
4	Ding et al., 2023	Y	Y	Y	Y	Y	Y	Y	Y	N	N	U	High
5	Huang et al., 2023	Y	Y	Y	Y	Y	Y	Y	Y	N	N	U	High
6	L. Wang et al., 2022	Y	Y	Y	Y	Y	Y	Y	Y	N	N	U	High
7	Denyes et al., 2017	Y	Y	Y	Y	Y	Y	Y	Y	N	N	U	High
8	Laube et al., 2014	Y	Y	N	Y	Y	Y	Y	Y	N	N	Y	Low
**Bacterial detection**													
9	Deng et al., 2024	Y	Y	Y	Y	Y	Y	Y	Y	N	N	Y	Hight
10	Hussain et al., 2024	Y	Y	Y	Y	Y	Y	Y	Y	N	N	Y	Hight
11	You et al., 2024	Y	Y	Y	Y	Y	Y	Y	Y	N	N	Y	High
12	X. Wang et al., 2024	Y	Y	Y	Y	Y	Y	Y	Y	N	N	Y	High
13	Zhang et al., 2023	Y	Y	Y	Y	U	Y	Y	Y	N	N	U	Moderate
14	Y. Wang et al., 2023	Y	Y	Y	Y	Y	Y	Y	Y	N	N	U	High
15	Ding, Zhu, et al., 2023	Y	Y	Y	Y	Y	Y	Y	Y	N	N	U	High
16	Lee et al., 2023	Y	Y	Y	Y	Y	Y	Y	Y	N	N	U	High
17	S. Wang et al., 2023	Y	Y	Y	Y	Y	Y	Y	Y	N	N	U	High
18	Lamas et al., 2023	Y	Y	Y	Y	Y	Y	Y	Y	N	N	U	High
19	J. Wang et al., 2022	Y	Y	Y	Y	Y	Y	Y	Y	N	N	U	High
20	Zhao et al., 2022	Y	Y	Y	Y	Y	Y	Y	Y	N	N	U	High
21	Huang et al., 2022	Y	Y	Y	Y	Y	Y	Y	Y	N	N	U	High
22	de Aquino et al., 2021	Y	Y	Y	Y	U	Y	Y	Y	N	N	U	Moderate
23	Hyeon et al., 2021	Y	Y	Y	Y	U	Y	Y	Y	N	N	U	Moderate
24	Ilhan et al., 2021	Y	Y	Y	Y	U	Y	Y	Y	N	N	U	Moderate
25	Nguyen et al., 2020	Y	Y	Y	Y	Y	Y	Y	Y	N	N	U	High

No.	Studies	Questions											Overall appraisal
		Q1*	Q2*	Q3*	Q4*	Q5	Q6	Q7	Q8	Q9	Q10	Q11	
25	Garrido-Maestu et al., 2019	Y	Y	Y	Y	Y	Y	Y	Y	N	N	U	High
27	Anany et al., 2018	Y	Y	Y	Y	U	Y	Y	Y	N	N	U	Moderate
28	F. Wang et al.,2017	Y	Y	Y	Y	U	Y	Y	Y	N	N	U	Moderate
29	Kim et al., 2014	Y	Y	Y	Y	U	Y	Y	Y	N	N	U	Moderate
30	Junillon et al., 2012	Y	Y	Y	Y	U	Y	Y	Y	N	N	U	Moderate
31	Li et al., 2010	Y	Y	Y	Y	U	Y	Y	Y	N	N	U	Moderate
32	Muldoon et al., 2007	Y	Y	Y	Y	Y	Y	Y	Y	N	N	U	High
33	Favrin et al., 2003	Y	Y	Y	Y	Y	Y	Y	U	N	N	U	Moderate
34	Chen et al., 1996	Y	Y	Y	Y	U	Y	Y	U	N	N	U	Moderate
35	Hirsh et al., 1983	Y	Y	Y	Y	U	Y	Y	U	N	N	U	Moderate

Critical question (*); yes (Y), no (N), unclear (U)

Criteria indicating low methodological quality included one or more "no" answers or "unclear" responses to critical questions, or three or more "no" answers to non-critical items. High methodological quality was determined when a study had a maximum of two "No" and one of "unclear" responses in non-critical items. Studies not meeting these criteria were considered to have moderate methodological quality.
